# Effect of Chiropractic Care on Auditory Attention in Healthy Individuals: An Analysis of the P300 Event-Related Potentials Component

**DOI:** 10.21315/mjms-10-2025-709

**Published:** 2026-02-28

**Authors:** Tahamina Begum, Courtney Bliese, Emily Drake, Margaret Sliwka, Stephanie Sullivan, Mohammed Faruque Reza

**Affiliations:** Dr. Sid E. Williams Center for Chiropractic Research, Life University, 1269 Barclay Cir, Marietta, Georgia 30060, USA

**Keywords:** event-related potential (ERP), chiropractic care, amplitude, latency, P300 ERP component, intervention

## Abstract

**Background:**

The effect of chiropractic care on auditory attention, as measured by event-related potentials (ERP), has not been examined in healthy young adults. This study aimed to assess the impact of a single chiropractic session on auditory attention by analysing the amplitude and latency of the P300 ERP component within the auditory oddball paradigm in healthy adults.

**Methods:**

Twenty-eight healthy adults participated in this chiropractic intervention study. ERP experiments were performed both before (pre-group) and one week after (post-group) a single chiropractic care session. During the experiments, a 64-channel electroencephalography (EEG)/ERP cap was used, and participants silently counted the target tone while disregarding standard tones. The amplitude and latency of the P300 ERP component were analysed using a standardised 10–20 EEG system across 19 electrode channels. A non-parametric Wilcoxon Signed-Rank Test (pairwise) was used to compare data groups.

**Results:**

The topographic map distribution revealed a widespread pattern during standard stimuli and a more localised pattern during target stimuli in the post-group than in the pre-group. Significantly higher P300 amplitudes were observed at Cz (*P* = 0.013) and O1 (*P* = 0.012), along with a significantly shorter P300 latency at P7 (*P* = 0.042) in the post-group than in the pre-group.

**Conclusion:**

Higher P300 amplitudes and shorter P300 latencies indicated improved attention. This evidence suggests that chiropractic care may enhance auditory attention in healthy individuals. However, its clinical importance remains uncertain. Therefore, it is premature to recommend chiropractic care as a treatment for boosting auditory attention.

## Introduction

In the human cognitive system, attention acts as a conductor guiding perception, regulating behaviour, and supporting learning. Traditionally, efforts to improve attention following interventions have focused on pharmacological and psychological methods. However, recent research suggests that chiropractic care, as a physical intervention, may significantly influence cognitive function. Chiropractic procedures focus on the spine and other joints, their relationship, and their effects on the nervous system. Chiropractic care can help relieve musculoskeletal issues that may affect the brain activity related to attentional control. Research has shown that chiropractic care can enhance attention by modulating activity in the prefrontal cortex (1) and increasing alpha- and beta-band power in the default mode network (2). Temporary changes in the brain function may occur after spinal manipulation (3). Functional neuroimaging and electroencephalography (EEG) studies have suggested that chiropractic care may influence sensorimotor integration and cortical processing (2), and potentially affect attention. Non-invasive EEG and somatosensory-evoked potential (SEP) methods are widely used to study brain connectivity, sensorimotor processing, and resting-state activity in both healthy individuals and patients with stroke, pain, Alzheimer’s, and Parkinson’s disease (4–7).

This study explores the emerging connection between chiropractic care and cognitive neuroscience by investigating whether chiropractic treatment influences auditory attention. The answer may be identified through neurophysiological measures, such as EEG, SEPs, and event-related potentials (ERPs). However, these results may differ depending on the method used. For instance, EEG provides data on alpha, beta, and gamma power; SEP measures N20/N30 amplitudes and latencies; and ERP assesses components such as P50, N100, N200, and P300. Early EEG and SEP findings suggest that chiropractic care may affect brain activity related to attention and executive function; however, its effect on ERP-measured attention performance in healthy adults remains largely unexplored.

Healthy individuals have participated in various studies that have examined the potential effects of chiropractic care. Healthy subjects showed a significant increase in bite force after a single chiropractic session (8). In healthy individuals, single-session chiropractic care has been shown to reduce experimentally induced pain. While Provencher et al. (6) found no changes in laser-evoked potential C2 and P2 components, Gevers-Montoro et al. (9) reported reduced capsaicin-induced pain with associated frontal high-gamma EEG activity following spinal manipulation. Studies using SEPs and resting-state EEG have shown that a single chiropractic session can alter cortical processing, particularly the N30 SEP amplitude, in healthy individuals (10–12), patients with stroke (4), and patients with Alzheimer’s and Parkinson’s disease (5). Although changes in connectivity (7) and N30 amplitude have been reported, no study has examined the effects of a single chiropractic session in healthy individuals using auditory ERP paradigms.

ERP measurement offers several advantages. It is a highly suitable, non-invasive tool for objectively measuring brain activity within certain cognitive domains (13). An ERP study offers the advantage of high temporal resolution and source localisation within 1 cm, is non-invasive (14), and has no conduction delay when recording postsynaptic potential (PSP) brain activity inside the skull. PSPs exhibit both positive and negative voltage deflections that appear as positive and negative ERP waveforms (13). ERP components represent specific neurocognitive processes; for example, the P300 shows a positive peak around 300 ms after the stimulus, with its amplitude reflecting attention and latency indicating processing speed (15). There are different categories of ERP components. For example, auditory stimuli can trigger the P50, MMN (N200), and P300 components, which are linked to various cognitive functions (16). ERP components are categorised as early (sensory/exogenous; peaks at ≤ 100 ms, e.g., C1, P50, N100) and late (cognitive/endogenous; peaks after 100 ms, e.g., N200, P300, N400) (14).

Both early and late ERP components can indicate deficits in neurocognitive processes, including synaptic dysfunction, making them useful for assessing and diagnosing Alzheimer’s disease (17, 18), mild cognitive impairment (19, 20), and traumatic brain injury or stroke (21–23). The P300 is the most frequently used ERP component for evaluating cognition, with higher amplitudes indicating greater attention and shorter latencies reflecting faster processing speeds (24). ERP amplitudes and latencies are typically measured using auditory or visual oddball paradigms, in which infrequent target stimuli elicit larger P300 amplitudes associated with attention, and latencies vary with task difficulty (25). Among auditory oddball tasks, P300 amplitudes are highest during silent counting of target tones compared to mental imagery or button-press conditions (26). One study found that the P300 amplitude and latency are unrelated, indicating that different biological mechanisms underlie abnormalities in the same information-processing domain (27). Therefore, the oddball paradigm is crucial in ERP research to analyse attentional and cognitive functions.

This study examined the effect of a single chiropractic care session on the auditory attention of healthy young individuals as assessed by the amplitude and latency of the P300 ERP component during an auditory oddball task in the ERP study.

## Methods

### Study Design

We focused on healthy participants who underwent a single chiropractic session. Therefore, this was an interventional study using a single chiropractic intervention in healthy adults (within-group design). There were two recordings of ERP experiments. ERP-1 was the baseline recording before adjustment (pre-group), followed by a single chiropractic session at the clinic by a chiropractor. ERP-2 was recorded one week after the session (post-group).

### Inclusion and Exclusion Criteria

The inclusion criteria were as follows: all participants were aged 18 to 40 years, able to tolerate wearing an EEG cap for 40 min; could sit still and quiet for 15 min; had no previous chiropractic care within two weeks before the experiments; and had no history of receiving other interventions such as osteopathic spinal manipulation, physical therapy, massage, body movement therapies, acupuncture, or similar treatments within two weeks prior to the start of the experiment.

We excluded participants who had severe mental disorders, such as schizophrenia or major depressive disorder; a history of head or spine injury or surgery within the last six months prior to the start of the experiment; or had used psychotropic medication (e.g. benzodiazepine, clonazepam, midazolam, etc.) in the two weeks before the experiment. Individuals with hearing impairment, current pregnancy, diagnosed osteoporosis, or joint instability were excluded from this study.

### Sample Size

The sample size was calculated for two dependent means using G*Power v3.1.9.7 (28, 29), assuming an effect size of 0.55, a two-sided significance level (α) of 0.05, and 80% power. This calculation indicated a minimum required sample size of 28 participants. After accounting for a 10% dropout rate, the final target sample size was 32 participants. A total of 28 participants completed the study and were included in the final analysis.

### Participant Recruitment, Experiment Location, and Consent

Participants were recruited through various channels, including e-mail announcements, word of mouth, social media, on-campus television screen presentations of the study flyer at Life University, and community flyer postings targeting students and staff at Life University in Marietta, Georgia, USA. The ERP experiments were conducted at the Center for Chiropractic Research (CCR), 1 Baltimore Place NW, Atlanta, GA 30308, USA. All participants provided written informed consent, in accordance with the principles outlined in the Declaration of Helsinki.

### Experimental Paradigm: Auditory Oddball Paradigm

The auditory oddball paradigm consists of two stimuli: a frequent standard tone and a rare target tone. The standard tones had a sound pressure level (SPL) of 20 dB, a high-frequency of repetition (80%), and a low-pitch (LP) (1 KHz). In contrast, the target tones were 20 dB SPL, low-frequency repetition (20%), and high-pitch (HP) (2 KHz). Both tones/stimuli lasted 200 ms with an inter-stimulus interval of 1.5 seconds. The stimuli were administered binaurally using noise-cancelling earphones.

### Experimental Procedure

E-Prime software (version 3.0; Psychology Software Tools Inc., Sharpsburg, PA, USA) was used to present the auditory oddball paradigm. All participants wore a 64-ERP net cap (64-channel Quik-cap hydronet) and silently counted the target tones while ignoring the standard tones. After completing the experiment, the examiners asked the participants how many target stimuli they had attended to ensure they paid attention during the task. One block of the ERP experiment included 160 standard tones and 40 target tones.

During data collection, an extra electrode was placed on the left cheek to monitor eye movements and blinking. The electrode impedance was kept below 50 kΩ throughout the experiment. The ERP cap is connected to a computer through an amplifier. The computer was equipped with the CURRY 8 software (Compumedics Neuroscan, Compumedics Limited, Melbourne, Australia) to record raw data for both offline and online analyses.

### Procedure of Chiropractic Care

Before the ERP experiment, participants underwent a case history and physical examination with a licensed chiropractor to confirm their eligibility for the study. The examination adhered to the standard of care established by the state’s chiropractic board requirements. It was performed by one of the coauthors, a licensed chiropractor with over 15 years of experience specialising in the Activator Method, also known as the Activator Protocol. Once it was confirmed that the participants showed no red flags or contraindications to treatment, they were allowed to proceed with the ERP experiments. Subsequently, the participants were placed in an adjustment room for chiropractic care using the Basic Activator Protocol. This protocol provides customised spinal adjustments based on a series of specific preset segmental tests to identify spinal levels that require adjustments (30). Adjustments were performed using an Activator V instrument (Activator Methods International Ltd., Phoenix, AZ, USA). This handheld device allows for the application of force within the range of “1” to “4.”

### Data Analysis

#### Raw Data Pre-processing

Raw data were pre-processed using MATLAB (MathWorks, Natick, MA, USA) (version R2024a), EEGLAB (31), ERPLAB (32), and E-Studio (33). During this process, the sampling rate was reduced from 1,000 Hz to 250 Hz. A basic finite-impulse-response bandpass filter (0.03 Hz to 30 Hz) was applied to the data. The number of electrode channels was reduced to 19 in the standard 10–20 system prior to interpolation. Bad channels, including those affected by eye movements, movement artefacts, ECG artefacts, and electrode issues, were identified by visual inspection of the data. These poor electrodes were interpolated using spherical interpolation methods. The interpolated data are subsequently referenced using an average reference montage. To identify and remove the artefacts caused by eye movements, blinks, cardiac activity, and muscle activity, we employed Infomax Independent Component Analysis (ICA) using the Picard algorithm in EEGLAB. Artefact components were classified and flagged based on the ICA results (31). The data were segmented into epochs from −200 ms to 800 ms, with a baseline correction applied to the −200 ms pre-stimulus interval. A difference waveform was created by subtracting the responses to the standard stimuli from those to the target stimuli. The amplitudes and latencies of the P300 ERP components were measured using the measurement tools in E-Studio (32, 33).

#### Data Post-processing (Statistical Analysis)

Statistical analyses were performed using the Statistical Package for the Social Sciences (SPSS) v28 to assess the significance of the differences between the pre- and post-groups. The Shapiro-Wilk test was used to check the normality of the data distribution. The results showed that P300 amplitudes and latencies across the 19 electrode channels were not normally distributed. Therefore, a non-parametric Wilcoxon Signed-Rank Test (paired) was conducted to evaluate the differences in P300 amplitudes and latencies between the pre- and post-intervention groups. Statistical significance was set at *P* < 0.05 for all comparisons. Effect size was estimated using r (Z/√N), following a published method (34), with values of 0.1, 0.3, and 0.5 indicating small, medium, and large effects, respectively.

## Results

### Demographic Information

This study recruited 28 healthy participants (14 males and 14 females), with a mean age of 27.55 years (± 4.04 SD).

### ERP Results

**Figure 1:**
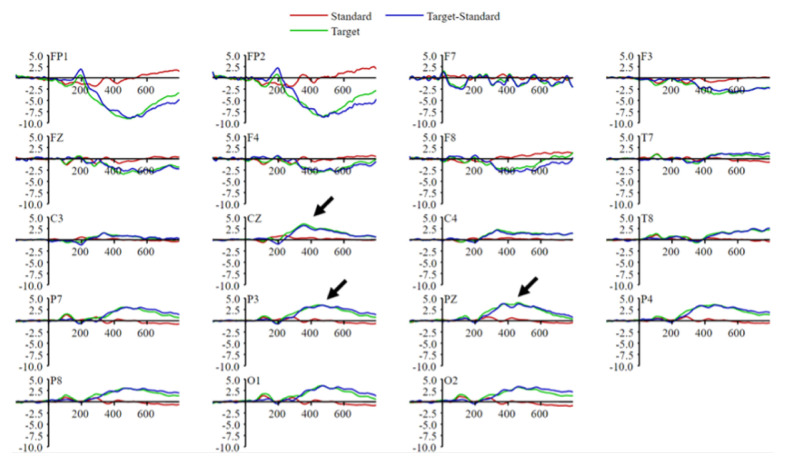
Grand average P300 ERP waveform in 28 healthy subjects before chiropractic adjustment (pre-group) across 19 electrode channels in the 10–20 system Red traces represent standard stimuli, green traces are target stimuli, and blue traces show the difference wave (target minus standard stimuli); The solid arrow indicates the evoked P300 ERP component

**Figure 2:**
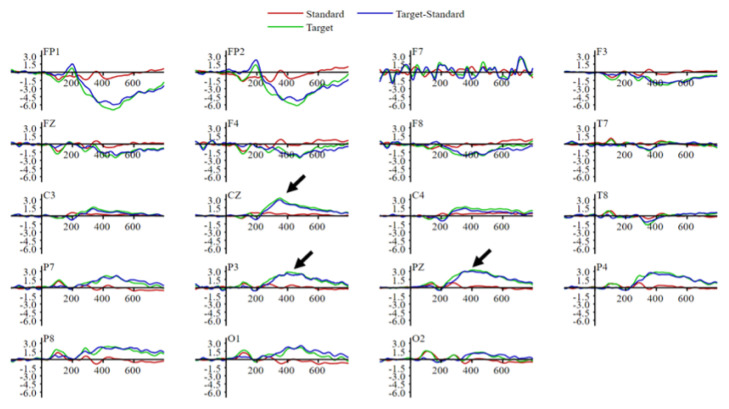
Grand average waveform of the P300 ERP component in 28 healthy subjects following chiropractic adjustment (post-group) across 19 electrode channels in the 10–20 system Red traces indicate standard stimuli, green traces are target stimuli, and blue traces show the difference wave (target minus standard stimuli); The solid arrow marks the evoked P300 ERP component

The grand average waveforms for the pre- and post-groups are shown in Figures 1 and 2, respectively, across the 19 electrode channels in the 10–20 system. The waveforms displayed traces of the standard and target stimuli, along with the difference wave (target minus standard) in the grand average depiction. In both figures, the solid arrows indicate the evoked P300 responses at different locations.

**Figure 3:**
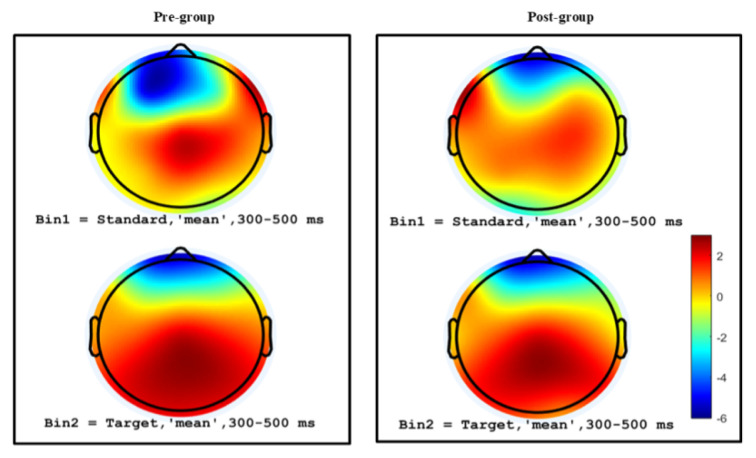
Distribution of topographic maps between pre- and post-groups within the 300–500 ms window

The brain topographic map within the 300–500 ms window (Figure 3) revealed that while the P300 topographic distribution was centred at Pz in both groups, notable differences were observed. For the standard stimuli, the distribution was more widespread, whereas for the target stimuli, it became more localised at the Pz in the post-group than in the pre-group (Figure 3).

Overall, the P300 amplitudes were higher in the post-intervention group than in the pre-intervention group at 12 out of the 19 electrode channels (Fp2, Fz, C3, Cz, T8, P7, P3, Pz, P4, P8, O1, and O2). Of the 12 channels examined, two (Cz and O1) showed significantly higher P300 amplitudes in the post-training group than in the pre-training group. At Cz, the post-group exhibited a median P300 amplitude of 3.67 μV (IQR: 0.92 μV), versus 2.80 μV (IQR: 1.78 μV) in the pre-group (*P* = 0.013, *r* = 0.469). Similarly, at O1, the post-group displayed a higher median P300 amplitude of 3.30 μV (IQR: 3.44 μV), compared to 2.43 μV (IQR: 1.44 μV) in the pre-group (*P* = 0.012, *r* = 0.474). Additionally, the two channels (Pz, *P* = 0.062; and P8, *P* = 0.062) showed a marginally significant increase in P300 amplitudes in the post-intervention group compared with the pre-intervention group. The other eight channels displayed a non-significant increase in P300 amplitude in the post-intervention group (Table 1). The same statistical analysis also showed that the post-group had a significantly shorter median P300 latency at the P7 location, 464 ms (IQR: 156 ms), compared to 482 ms (IQR: 90 ms) in the pre-group (*P* = 0.042, *r* = 0.385) (Table 1). The effect size analysis showed moderate effects for P300 amplitude (*r* = 0.469 and *r* = 0.474) and a small-to-moderate effect for P300 latency (*r* = 0.385), indicating significant post-intervention changes.

## Discussion

This study investigated the impact of a single chiropractic intervention on auditory attention in healthy individuals using the auditory oddball paradigm with a focus on the amplitudes and latencies of the P300 ERP component. Following a single chiropractic session, significantly higher P300 amplitudes with moderate effect sizes were observed at two locations, and significantly shorter latencies with small-to-moderate effect sizes were observed at a different location. These objective findings suggest that chiropractic care may improve auditory attention in healthy individuals, highlighting its potential to enhance cognitive function, particularly auditory attention, across diverse patient groups.

The P300 ERP component is a well-known neural marker used in cognitive neuroscience to evaluate attention, working memory, and decision-making. Many studies focusing on the P300 have examined its amplitude and latency, which are influenced by cognitive states and environmental factors. The literature shows either simultaneous changes in both the P300 amplitude and latency or changes in only one of them. The most accepted interpretation is that a larger amplitude and shorter latency of the P300 ERP component in the auditory oddball paradigm indicate better cognitive performance or increased auditory attention (24). An extended P300 latency indicates a longer processing time for auditory information (35). Patients with mild cognitive impairment have increased P300 latency because they require more cognitive effort to process target stimuli (36). The shorter amplitude and longer latency of the P300 component have been observed in patients with mild cognitive impairment (37), spinocerebellar ataxia type 2 (SCA2) (38), increased age (39), and Alzheimer’s disease (17) due to cognitive decline, indicating a lack of attention. Significantly higher P300 amplitudes were observed in pregnant women during the auditory oddball paradigm, with no notable changes in P300 latency, suggesting that pregnant women exhibit greater auditory attention (40). Furthermore, significantly higher P300 amplitudes without any notable changes in P300 latency were observed in patients with mild brain injury (MBI) seven days and two months after a road traffic accident. This suggests that patients with MBI paid more attention to identifying the target stimuli than the control group (41, 42). However, this study found both significantly higher P300 amplitudes (Cz and O1) and significantly shorter P300 latencies (P7) after chiropractic intervention in healthy young adults. These results are consistent with those of other studies (24, 40, 42) and suggest that chiropractic care may improve auditory attention in healthy individuals.

Few studies have examined the improvements in cognitive function after various interventions. A single chiropractic care session in patients with MBI resulted in significantly higher Stroop test scores, improved gaze stability during vestibulo-ocular reflex testing, and better fixation stability with egocentric localisation. These results contributed to improvements in visual and cognitive function following chiropractic treatment via spinal and proprioceptive mechanisms (41). Five weeks of intensive domain-general adaptive working memory training resulted in larger training gains in both young and older adults (43). The therapeutic effect of repetitive transcranial magnetic stimulation (rTMS) over the unaffected hemisphere in stroke patients has been demonstrated to result in a higher visual P300 amplitude, suggesting that cognitive compensation in the unaffected hemisphere may play a key role in enhancing visuospatial neglect (VSN) (44). Another rTMS intervention study demonstrated a significant increase in the P300 amplitude and shorter P300 latency in patients with stroke, indicating cognitive improvement (45). Advanced rehabilitation combining translingual neurostimulation (TLNS) and physiotherapy can improve visual attention, as evidenced by a significant increase in the N100 and N200 amplitudes after treatment (46). Cognitive behavioural therapy has been shown to significantly increase the P300 amplitude in patients with Tourette’s disorder and improve their condition (47). There is a notable lack of research on the effects of chiropractic interventions in healthy populations, especially regarding objective markers of amplitude and latency of the P300 ERP component. This study addresses this gap for the first time, demonstrating that chiropractic treatment may enhance auditory attention, as evidenced by significantly higher P300 amplitude and shorter P300 latency after the intervention. The moderate effect sizes observed for P300 amplitude and the small-to-moderate effect sizes for P300 latency suggested that the intervention had a noticeable impact on attentional resource allocation and cognitive processing speed. Research focusing on the P300 ERP component in relation to chiropractic care can provide valuable insights into how various treatments or experiences affect neural function. Further studies are necessary to confirm and better understand the connection between chiropractic care and P300 ERP components.

### Limitations of This Study

This study has several limitations, including a small sample size and a single-group design, which reduce statistical power, hinder causal inference, and limit generalizability. Without a control group, observed changes cannot be attributed with certainty to the intervention. Nonetheless, this study was designed as an exploratory investigation and provides feasibility data to inform future controlled trials.

## Conclusion

This study aimed to investigate the effects of chiropractic care on auditory attention in healthy adults by analysing the amplitude and latency of the P300 ERP component. The results showed significantly increased P300 amplitudes with a moderate effect size and decreased P300 latency with a small-to-moderate effect size, indicating that auditory attention may improve following chiropractic treatment. Additionally, the P300 ERP component functions as a valid objective biomarker for assessing cognitive function during chiropractic sessions. This study highlights the potential of chiropractic care to enhance cognitive performance and encourages further studies to investigate its benefits across various patient populations.

## Figures and Tables

**Table 1:** P300 amplitudes (μV) and latencies (ms) for pre- and post-groups, including *P*-value (effect size, *r*-value)

Areas	P300 amplitudes (μV)		P300 latencies (ms)		*P*-value (effect size, *r*-value)	

	Pre-group, median (IQR)	Post-group, median (IQR)	Pre-group, median (IQR)	Post-group, median (IQR)	P300 amplitude	P300 latency
FP1	2.71 (0.39)	2.44 (2.13)	409 (69)	352 (229)	0.466 (0.138)	0.764 (0.057)
FP2	2.43 (0.84)	2.56 (3.08)	394 (107)	452 (266)	0.539 (0.116)	0.121 (0.293)
F7	1.72 (1.79)	1.35 (1.31)	502 (142)	478 (199)	0.425 (0.151)	0.381 (0.166)
F3	1.27 (2.87)	1.69 (2.17)	348 (164)	344 (180)	0.682 (0.078)	0.866 (0.032)
Fz	2.20 (2.07)	2.07 (3.03)	386 (152)	356 (134)	0.399 (0.159)	0.718 (0.068)
F4	2.49 (2.92)	2.02 (1.91)	412 (223)	384 (139)	0.327 (0.185)	0.327 (0.185)
F8	2.83 (3.66)	1.79 (0.94)	420 (153)	462 (224)	0.127 (0.288)	0.264 (0.211)
T7	2.38 (2.28)	2.06 (2.16)	532 (112)	492 (128)	0.452 (0.142)	0.198 (0.243)
C3	2.53 (2.20)	2.68 (1.78)	356 (198)	392 (174)	0.295 (0.198)	0.799 (0.048)
CZ	2.80 (1.78)	3.67 (0.92)	356 (124)	362 (132)	0.013 (0.469)	0.559 (0.110)
C4	2.35 (2.53)	2.69 (2.54)	392 (197)	360 (145)	0.855 (0.034)	0.264 (0.211)
T8	2.17 (2.19)	2.25 (1.88)	494 (144)	500 (127)	0.802 (0.047)	0.855 (0.034)
P7	2.44 (1.48)	2.79 (2.31)	482 (90)	464 (156)	0.274 (0.207)	0.042 (0.385)
P3	3.06 (1.53)	3.42 (3.03)	460 (116)	452 (125)	0.194 (0.245)	0.592 (0.101)
PZ	2.93 (1.10)	3.45 (1.41)	448 (103)	398 (121)	0.062 (0.353)	0.155 (0.269)
P4	3.21 (2.42)	3.38 (2.15)	450 (130)	426 (172)	0.210 (0.237)	0.839 (0.038)
P8	2.95 (2.06)	3.62 (1.92)	478 (106)	492 (167)	0.062 (0.353)	0.933 (0.016)
O1	2.43 (1.44)	3.30 (3.44)	470 (122)	470 (126)	0.012 (0.474)	0.973 (0.006)
O2	2.93 (2.63)	3.30 (2.29)	478 (105)	458 (133)	0.096 (0.314)	0.239 (0.223)

IQR = interquartile range
